# Clinical Significance of Expression Changes and Promoter Methylation of PLA2R1 in Tissues of Breast Cancer Patients

**DOI:** 10.3390/ijms21155453

**Published:** 2020-07-30

**Authors:** Noha Mitwally, Einas Yousef, Ahmad Abd Al Aziz, Mohamed Taha

**Affiliations:** 1 Department of Biochemistry, Faculty of Pharmacy, Cairo University, Cairo 11562, Egypt; nmtwally@dau.edu.sa; 2 Basic Medical Sciences Department, College of Medicine, Dar Al Uloom University, Riyadh 7222, Saudi Arabia; enasesawy@gmail.com; 3 Histology Department, Faculty of Medicine, Menoufia University, Shebin Elkom 3251, Egypt; 4 Surgery Department, Faculty of Medicine, Kasr Al Ainy Hospital, Cairo University, Cairo 11562, Egypt; zizoo_81@hotmail.com

**Keywords:** PLA2R1, breast cancer, TNBC, promoter methylation, bioinformatics analysis

## Abstract

Phospholipase A2 receptor 1 (PLA2R1) expression and its role in the initiation and progression of breast cancer are an unresolved issue. PLA2R1 was found to endorse several tumor suppressive responses, including cellular senescence and apoptosis. Previous in vitro studies demonstrated that DNA hypermethylation was highly associated with the epigenetic silencing of *PLA2R1* in breast cancer cell lines. Our objective was to study the level of PLA2R1 mRNA expression and the methylation of its promoter in different histological grades and molecular subtypes of breast cancer. We performed bioinformatics analyses on available human breast cancer expression datasets to assess the PLA2R1 mRNA expression. We used qRT-PCR to evaluate the PLA2R1 mRNA expression and its promoter’s methylation in breast cancer tissue in comparison to breast fibroadenomas. Our results describe, for the first time, the expression of PLA2R1 and the methylation of its promoter in human breast cancer tissues. A significant downregulation of PLA2R1, together with hypermethylation of the promoter was detected in breast cancers of different histological grades and molecular subtypes when compared to benign breast tissues. *PLA2R1* promoter hypermethylation was associated with aggressive subtypes of breast cancer. In conclusion, *PLA2R1* promoter hypermethylation is a potentially useful diagnostic and prognostic biomarker and could serve as a possible therapeutic target in breast cancer.

## 1. Introduction

Phospholipase A2 receptor 1 (PLA2R1) is a type I transmembrane receptor that belongs to the mannose receptor family and exists in both a transmembrane and a soluble form [1]. The physiological function of human PLA2R1 is related to its distinct affinities to interact with several secretory phospholipase A2 (sPLA2) proteins, various types of carbohydrates and collagens [2]. Although its precise function is not clear, its binding to sPLA2 induces both positive and negative regulation in diverse signaling pathways, including cell proliferation, growth, differentiation and apoptosis [3]. Aberrant expression of PLA2R1 has been associated with the development and progression of different types of cancer. Elevated levels of PLA2R1 are associated with pancreatic, gastric, prostate and ovarian carcinomas, while it is downregulated in leukemia, kidney, thyroid and breast carcinomas [3,4,5,6,7,8].

There is now a compelling body of evidence suggesting that alterations to epigenetic markers, such as DNA methylation, histone modification and posttranscriptional gene regulations by microRNAs, are commonly noticed in the development and progression of different types of cancer [9]. Mechanistically, cancer cells endorse *PLA2R1* promoter hypermethylation to diminish its tumor suppressive effects, enhancing tumorigenesis [4]. *PLA2R1* promoter hypermethylation was detected in leukemic cells, renal cell carcinoma and breast cancer cell lines [4,6,10,11]. Assessing promoter methylation as an epigenetic regulator that is associated with the differential expression of PLA2R1 in breast cancer may potentially define novel therapeutic targets, diagnostic and/or prognostic biomarkers.

Breast cancer is the most frequently diagnosed cancer in women worldwide with an estimated 2.1 million new cases each year [12]. Despite an apparently similar phenotype, mammary tumors are highly heterogeneous, embracing a group of genetically and epigenetically distinct diseases with variable clinical courses, histopathological features, molecular subtypes and various responses to treatment [13]. Gene expression profiling has paved the way for a more comprehensive classification of breast cancers, allowing their separation into robust molecular entities. The molecular subtyping of breast cancers plays a critical role in predicting the biologic behavior of breast malignancies and for developing more effective therapeutic approaches [14]. Despite the success of current therapies, we still need to uncover unique genetic alterations or tumor characteristics that might be translated into diagnostic and prognostic biomarkers or pharmacologically amenable targets.

To our knowledge, this is the first study to examine the expression of PLA2R1and its promoter’s methylation, as one of epigenetic regulators, in human breast cancer tissues. Our goal was to study the level of PLA2R1 mRNA expression in different histological grades and molecular subtypes of breast cancer. More specifically, we wanted to assess the degree of *PLA2R1* promoter methylation that may regulate the expression of PLA2R1 in human breast cancer tissues. In the present study, we showed that significant downregulation of PLA2R1 was detected in breast cancers of different histological grades and molecular subtypes when compared to benign breast tissues. Our results indicated that *PLA2R1* promoter hypermethylation was not only inversely correlated with PLA2R1 downregulation but also associated with the most aggressive subtypes of breast cancers. Hence, we can conclude that *PLA2R1* promoter hypermethylation is a potential diagnostic and prognostic biomarker in breast cancer.

## 2. Results

### 2.1. Downregulation of PLA2R1 mRNA Expression Is Associated with High Histological-Grade Breast Cancer

**Figure 1:**
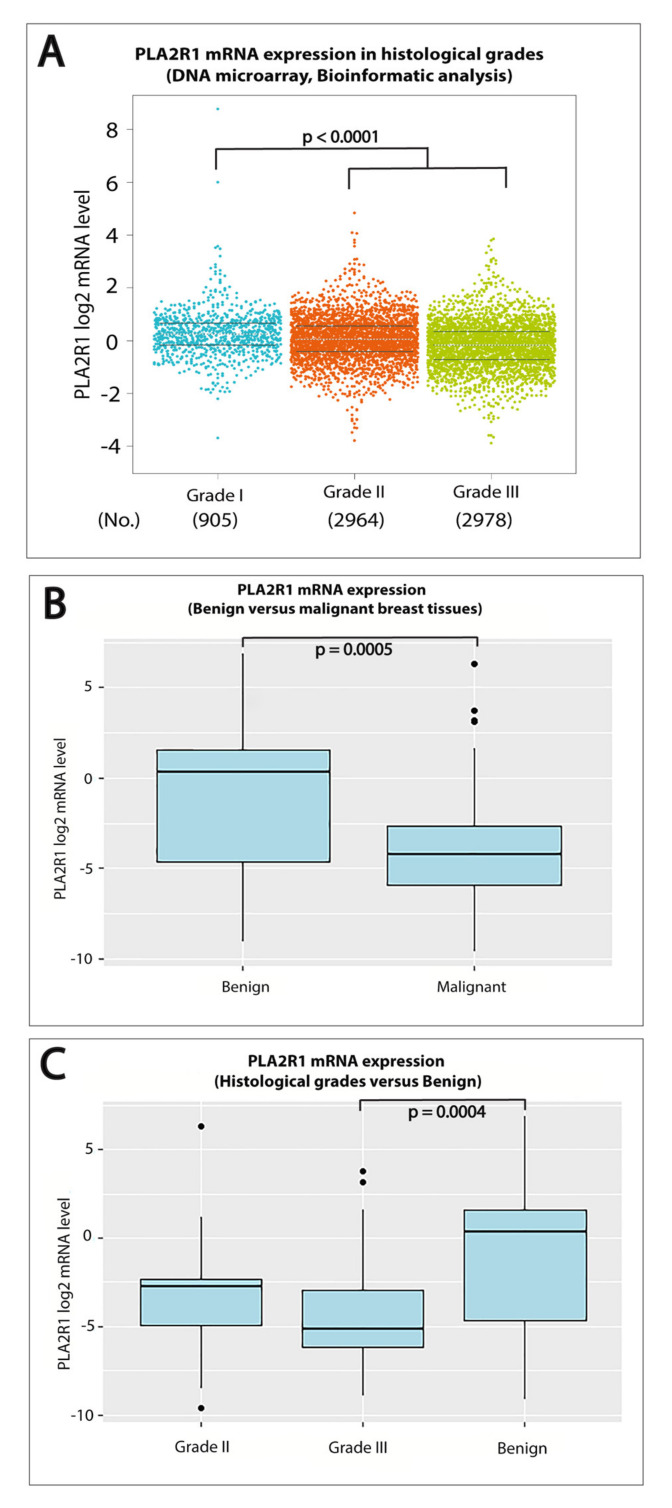
Differential expression of PLA2R1 among different histological grades of breast cancer compared to benign fibroadenomas. (**A**) Boxplots illustrating significantly lower expression levels of PLA2R1 mRNA in Grade II and III in comparison to Grade I breast cancer (*p* < 0.0001) from the bc-GenExMiner DNA microarray database. (**B**) Levels of PLA2R1 mRNA were measured using RT-qPCR in benign and malignant breast tumors and normalized to GAPDH mRNA as reference gene. Boxplots showing the downregulation of PLA2R1 mRNA expression in malignant breast cancer tissues compared to benign controls (*p* = 0.0005). (**C**) Boxplots depicting a significantly lower expression level of PLA2R1 mRNA in Grade III breast cancer compared to the benign controls (*p* = 0.0004).

To explore the potential role of PLA2R1 in breast cancer tumorigenesis, we evaluated the expression of PLA2R1 mRNA. The web application bc-GenExMiner database version v4.4 [15] was used to study the differential expression of PLA2R1 mRNA between various histological grades of DNA microarray and RNAseq datasets comprising 10,001 and 4712 breast cancer patients. Based on the analysis of these datasets, we detected a significant decrease (*p* < 0.0001) in the mRNA level of PLA2R1 in Grade II and III in comparison to Grade I breast cancer (Figure 1A).

The PLA2R1 mRNA expression levels were next assessed in both benign and malignant breast tissue samples using qRT-PCR. A statistically significant lower level of PLA2R1 mRNA expression (fold change = 0.052, *p* = 0.0005) was detected in breast cancer tissues compared to benign control (Figure 1B). To validate the results obtained from our bioinformatics analysis, we assessed the mRNA expression of PLA2R1 in different histological grades of human breast cancer (Table 1). Our findings confirmed that lower levels of PLA2R1 mRNA expression were detected in Grade II (fold change = 0.15) and Grade III (fold change = 0.028) breast cancer when compared to the benign control. However, only high histological-grade (Grade III) breast cancer reached a statistically significant level (*p* = 0.0004) when compared to benign breast tissue (Figure 1C).

### 2.2. Lower Levels of PLA2R1 mRNA Expression Were Significantly Associated with Triple Negative Breast Cancers (TNBC)

**Figure 2:**
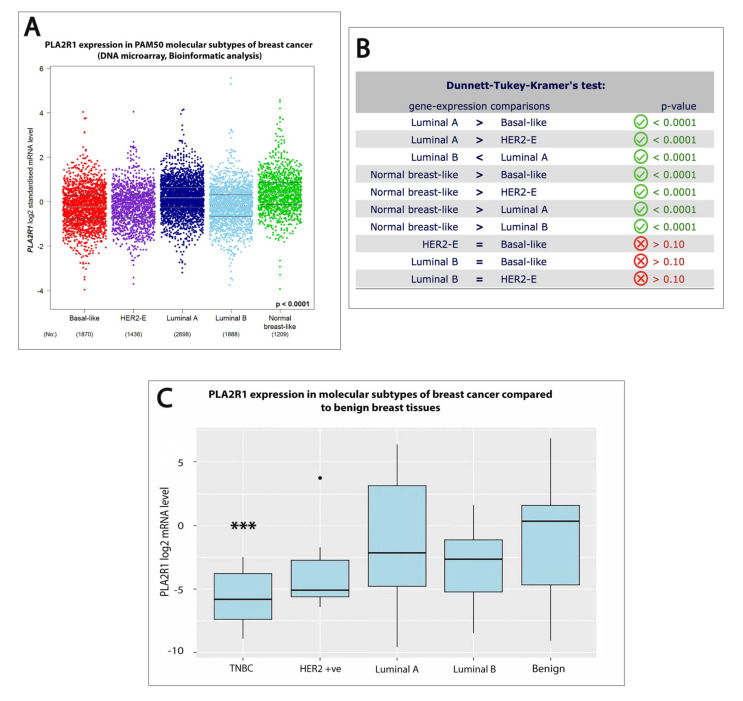
Lower level of PLA2R1 mRNA expression was significantly associated with Triple Negative Breast Cancers (TNBC). (**A**) Boxplots displaying lower expressions of PLA2R1 mRNA levels in basal-like, luminal B and HER2-positive breast cancers compared to luminal A and normal breast-like cancers (*p* < 0.0001) based on the bioinformatics analysis from the bc-GenExMiner DNA microarray database. (**B**) *p* values generated by Dunnett–Tukey–Kramer’s test to evaluate the difference in gene expressions among distinct molecular subtypes retrieved from bc-GenExMiner DNA microarray database. (**C**) Boxplots demonstrating lower expression levels of PLA2R1 mRNA in different molecular subtypes of breast cancer compared to benign breast fibroadenomas, however, only TNBC subtype reaches statistical significance level (*p* = 0.0002). *** indicate significant difference in comparison to benign control.

We further explored whether PLA2R1 mRNA downregulation was associated with different molecular subtypes of breast cancer. Our bioinformatics analysis from the bc-GenExMiner database demonstrated a lower expression of PLA2R1 mRNA in basal-like, human epidermal growth factor receptor 2 (HER2)-positive and luminal B breast cancers in comparison to both luminal A and normal breast-like cancers (*p* < 0.0001) (Figure 2A,B) [15]. To substantiate the data obtained from the bioinformatics analysis, PLA2R1 mRNA expression was then evaluated in breast cancer tissues of different molecular subtypes. The molecular subtypes were defined using the expression of the following surrogate immunohistochemical markers—estrogen receptor (ER), progesterone receptor (PR), HER2 and Ki-67 (Table 1) [16,17]. As depicted in Figure 2C, the PLA2R1 expression was significantly downregulated in TNBC (fold change = 0.017, *p* = 0.0002) compared to benign control tissues. Although the HER2-positive, luminal A and luminal B subtypes of breast cancer demonstrated lower expression of PLA2R1 mRNA with median fold changes (0.029, 0.232 and 0.158, respectively), none of these differences approached statistical significance. Our data indicated that the differential expression of PLA2R1 reflected the degree of differentiation of breast cancer cells, which supports its clinical usefulness.

### 2.3. PLA2R1 Promoter Hypermethylation Was Associated with Aggressive Subtypes of Breast Cancer

**Figure 3:**
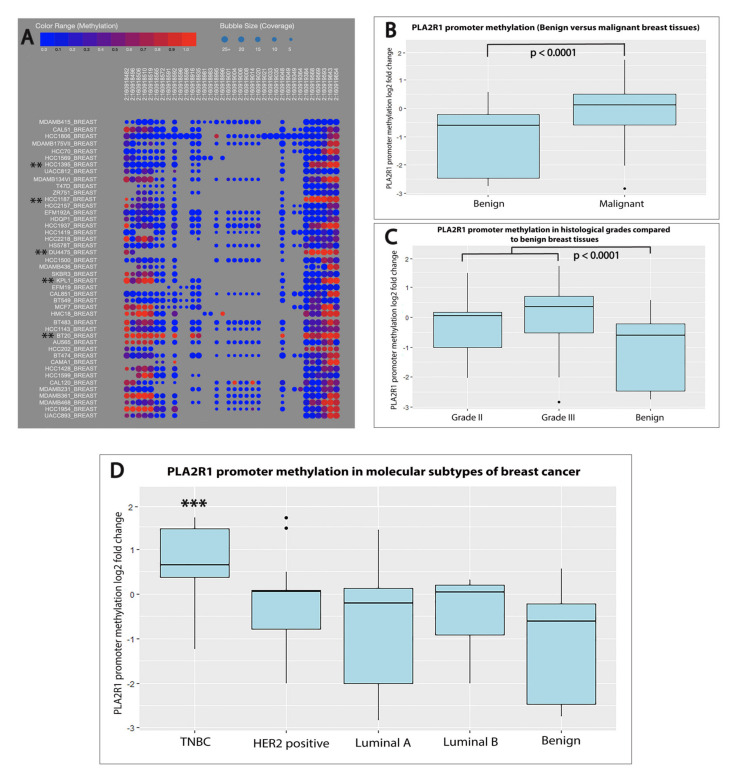
*PLA2R1* promoter methylation in different grades and molecular subtypes of breast cancer compared to mammary fibroadenomas. (**A**) A bubble map created by the Cancer Cell Line Encyclopedia (CCLE) showing *PLA2R1* methylation in 44 breast cancer cell lines. ** indicate the TNBC cell lines. (**B**) Boxplots depicting the hypermethylation of the *PLA2R1* promoter in breast cancer tissues compared to breast fibroadenomas as control (*p* < 0.0001). (**C**) Boxplots showing significant hypermethylation of the *PLA2R1* promoter in Grade II (*p* = 0.014) and Grade III (*p* < 0.0001) breast cancer compared to benign control tissues. (**D**) Boxplots showing that methylation of the *PLA2R1* promoter was significantly elevated (*p* < 0.0001) in TNBC when compared with benign breast fibroadenomas. *** indicate significant difference in comparison to benign control.

We sought to check if the suppressed expression of PLA2R1 mRNA was associated with a specific methylation pattern of its promoter. We performed a bioinformatics analysis on the publicly available Cancer Cell Line Encyclopedia (CCLE) to assess *PLA2R1* CpG island methylation in 44 breast cancer cell lines of different molecular subtypes [18]. As shown in Figure 3A, hypermethylation of the *PLA2R1* promoter was detected in triple negative (HCC1395, HCC1187, DU4475, KPL1 and BT20) and HER2-positive (SKBR3, HCC202 and HCC1954) breast cancer cell lines [19]. The MCF7, HMC18, MDA-MB-361 and BT483 cell lines were the only luminal cell lines in this analysis that revealed hypermethylation of the *PLA2R1* promoter [19,20,21].

Next, we assessed if the *PLA2R1* promoter average methylation level was associated with decreased PLA2R1 mRNA expression in human breast cancer tissues. Using qRT-PCR, we found that methylation of the *PLA2R1* promoter was significantly elevated (fold change = 1.079, *p* < 0.0001) in breast cancer tissues when compared with the benign control (Figure 3B). A negative correlation was detected between the expression of PLA2R1 mRNA and the levels of *PLA2R1* promoter methylation (*r* =− 0.2). A statistically significant hypermethylation of the *PLA2R1* promoter was detected in both Grade II (fold change = 1.034, *p* < 0.0001) and Grade III (fold change = 1.279, *p* < 0.0001) breast cancers compared to benign breast tissues (Figure 3C).

In further agreement with the cell line bioinformatics analysis, methylation of the *PLA2R1* promoter was significantly elevated (fold change = 1.562, *p* < 0.0001) in TNBC when compared with benign breast fibroadenomas. Non-significant hypermethylation of *PLA2R1* promoter was detected in HER2-positive (fold change= 1.033, *p* = 0.07), luminal A (fold change = 0.871, *p* = 0.5) and luminal B (fold change =1.03, *p* = 0.5) breast cancers (Figure 3D). We next sought to compare the level of *PLA2R1* promoter methylation in TNBC with its level in the HER2-positive, luminal A and luminal B molecular subtypes. We found that methylation of the *PLA2R1* promoter was significantly elevated in TNBC tissues compared to HER2-positive, luminal A and luminal B types (*p* = 0.002, *p* = 0.004 and *p* = 0.02), respectively.

### 2.4. PLA2R1 Promoter Methylation Outperformed PLA2R1 Expression as a Diagnostic and Prognostic Marker of Breast Cancer

**Figure 4:**
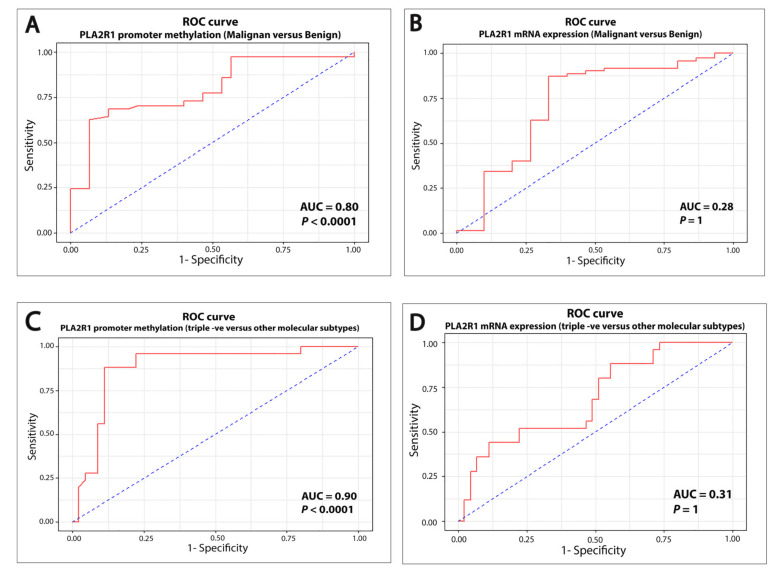
*PLA2R1* promoter methylation discriminated two distinct subgroups of breast cancer based on ROC curve analysis. (**A**,**B**) ROC curve analysis demonstrating that *PLA2R1* promoter methylation was more accurate than PLA2R1 expression in discriminating breast cancer from benign fibroadenomas. (**C**,**D**) ROC curve analysis demonstrating that *PLA2R1* promoter methylation surpassed PLA2R1 expression in discriminating TNBC tumors from other molecular subtypes of breast cancer.

Our results indicated that both PLA2R1 expression and its promoter methylation could be considered as potential diagnostic biomarkers in breast cancer as both can separate out benign from malignant breast tissues. We further evaluated the diagnostic accuracy of both markers using receiver operating characteristic (ROC) curve analysis. Our results showed that *PLA2R1* average promoter methylation was able to distinguish breast cancer from a benign control with the area under the curve (AUC) = 0.80 (*p* < 0.0001) and the sensitivity, specificity and cutoff point at 63%, 93% and 1.03, respectively (Figure 4A). Of note, the cutoff point of 1.03 corresponds to 32.5% of the average promoter methylation. When ROC analysis was conducted for PLA2R1 expression, we found the AUC = 0.28 (*p* = 1) and the sensitivity, specificity and cutoff point at 99%, 10% and 0, respectively (Figure 4B). Taken together, these results imply that *PLA2R1* promoter methylation outperformed PLA2R1 expression in discriminating breast cancer from benign fibroadenomas.

We further conducted ROC analysis to examine the ability of both markers in differentiating the TNBC tumors from the other breast cancer molecular subtypes (HER2-positive, luminal A and luminal B). As depicted in Figure 4C, *PLA2R1* promoter methylation can distinguish the TNBC tumors from HER2-positive, luminal A and luminal B subtypes with the AUC = 0.9 (*p* < 0.0001) and the sensitivity, specificity and cutoff point at 88%, 89% and 1.27, respectively. The cutoff point of 1.27 corresponds to 28% of the average promoter methylation. When ROC analysis was used to assess the PLA2R1 expression in discriminating the TNBC from other molecular subtypes, we found the AUC = 0.31 (*p* = 1) with the sensitivity, specificity and cutoff point at 100%, 2% and 0, respectively. These results suggest that *PAL2R1* promoter methylation is a potentially useful prognostic marker as it can discriminate TNBC from other molecular subtypes of breast cancer.

## 3. Discussion

PLA2R1 expression and its role in the tumorigenesis of breast cancer are still not completely understood. In the present study, we explored the expression of PLA2R1 in breast cancer tissues of different histological grades and molecular subtypes in comparison to benign mammary tumors. We also assessed the degree of *PLA2R1* promoter methylation in relation to the expression of PLA2R1 in human breast cancer tissues. Our findings revealed that PLA2R1 was differentially expressed among different histological grades and molecular subtypes of breast cancers compared to benign ones. However, only the aggressive breast cancers—Grade III and the TNBC subtype—demonstrated statistically significant downregulation of PLA2R1 mRNA expression compared to benign control.

**Figure 5:**
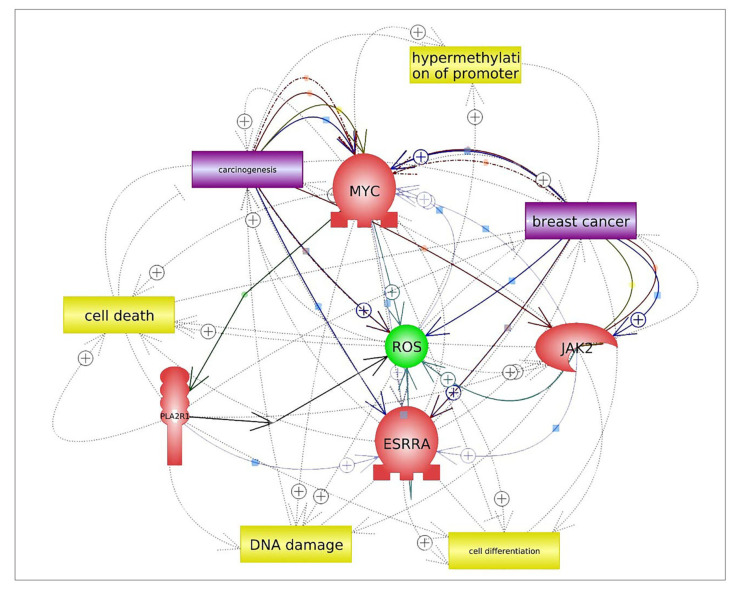
Pathway studio network analysis of *PLA2R1* and *PLA2R1* promoter methylation in breast cancer [26]. The created biological network demonstrated that *PLA2R1* was involved in several vital biological processes of breast cancer, which included triggering DNA damage, carcinogenesis, cell death and cell differentiation.

Previous in vitro studies reported low expression of PLA2R1 mRNA in different types of cancer, such as leukemia, renal, thyroid and breast cancers [3,5,22]. However, this was clearly at odds with the observations of another study that reported significant upregulation of PLA2R1 in prostate cancer when compared to normal tissues [23]. The fact that PLA2R1 mRNA was observed at low levels in breast cancer strongly supports its potential tumor suppressor role. This is in line with previous findings by others who reported that PLA2R1 was shown to regulate several anti-tumor and anti-inflammatory responses, including proliferation, cell transformation, apoptosis and senescence in breast cancer [22,24,25]. Although we have not directly addressed the question of how *PLA2R1* suppresses tumorigenesis in breast cancer, there are many possible mechanistic explanations that can be underlined. *PLA2R1* is involved in several vital biological process of breast cancer, which include triggering DNA damage, carcinogenesis, cell death and cell differentiation (Figure 5) [26]. One conceivable mechanism is that PLA2R1 activates the Janus kinase 2 (JAK2) pathway as well as induces estrogen-related receptor alpha1 (ESRRA). Both pathways direct their activities towards tumor suppression by accumulating reactive oxygen species (ROS), which impacts the mitochondrial biology, leading to senescence and apoptosis [5,22,27]. Another possible explanation is that PLA2R1 triggers DNA damage through the activation of the p53 signaling molecule—one of its downstream targets [24].

The disruption of epigenetic mechanisms plays a crucial role in the neoplastic cellular transformation essential for cancer initiation and progression. Aberrant DNA methylation, histone modification, as well as posttranscriptional gene regulations by microRNAs were previously detected at earlier stages of malignant cellular transformation [28,29,30]. We decided to investigate the DNA methylation of the *PLA2R1* promoter as an epigenetic regulator that might target PLA2R1 mRNA expression. Our results demonstrated a significant hypermethylation of the *PLA2R1* promoter in breast cancer tissues compared to the benign controls. This finding was consistent with reports of the association between the hypermethylation of the *PLA2R1* promoter and the loss of its expression in breast cancer [3,10]. This detected hypermethylation of the *PLA2R1* promoter may explain the downregulation of PLA2R1 mRNA expression in breast cancer tissues. Our results suggest that breast cancer cells may use the hypermethylation of the *PLA2R1* promoter to induce its downregulation as a defense mechanism against its tumor suppressive effects. Previous in vitro studies explained the mechanism of the *PLA2R1* promoter hypermethylation in various types of cancer [4,6,23]. As depicted in Figure 5, hypermethylation of the *PLA2R1* promoter in breast cancer is triggered by cellular myelocytomatosis (c-MYC)-mediated promoter methylation. The binding of c-MYC to the *PLA2R1* promoter induces *PLA2R1* DNA methylation through the recruitment of DNA methyl transferase, which results in the suppression of its expression [3,6,31].

We also found that *PLA2R1* promoter hypermethylation was significantly associated with TNBC, which are well known for aggressive behavior. This observation is supported by another study that also reported the association of *PLA2R1* promoter hypermethylation with TNBC cell lines [10]. Together with previously published data, the results presented herein clearly underscore the role of DNA methylation in *PLA2R1* gene regulation in mammary tumors. The significant association of *PLA2R1* promoter hypermethylation with TNBC implies its usefulness as a potential prognostic marker in breast cancer.

Our study, to our knowledge, is the first to assess the expression of PLA2R1 and its promoter methylation as one of its epigenetic regulators in human breast cancer tissues. However, the intrinsic limitation of this study is the small sample size that did not offer adequate power to study the relationships between the epigenetic factors and the downregulation of PLA2R1. Large-scale clinical studies are required to assess the prognostic relevance of PLA2R1 and its promoter methylation to breast cancer outcomes and treatment responses.

In conclusion, our study suggests that *PLA2R1* promoter methylation is a potentially useful diagnostic and prognostic biomarker in breast cancer. This sheds light on the role of promoter methylation as an epigenetic regulator of PLA2R1 expression in breast cancer. We can also anticipate that *PLA2R1* promoter methylation might serve as a potential therapeutic target in breast cancer. These novel findings on the epigenetic control of *PLA2R1* and its link to the aggressive subtypes of mammary gland carcinomas open the door to new areas of research in cancer biology.

## 4. Materials and Methods

### 4.1. Bioinformatics Analysis

The web application bc-GenExMiner database version v4.4 [15] was used to study the differential expression of PLA2R1 mRNA among various histological grades and PAM50 molecular subtypes of breast cancer. This application includes DNA microarray and RNAseq datasets comprising 10,001 and 4712 breast cancer patients, respectively. Welch’s test and Dunnett–Tukey–Kramer’s test were conducted to evaluate the difference in gene expressions among distinct population subgroups.

We have used the publicly available CCLE (https://portals.broadinstitute.org/ccle) to assess the *PLA2R1* gene methylation in 44 breast cancer cell lines [18]. Gene methylation can be visualized through a bubble map where the X-axis displays the position of the methylation data (the number before the colon is the chromosome and the number after the colon is the position of methylation) and the Y-axis displays the name of breast cancer cell lines in which the methylation was measured. In the bubble map, the bubble size represents the coverage, its color represents methylation, with warmer colors being more methylated [18]. We used the publicly available Pathway Studio Web (Elsevier, Netherlands) (https://mammalcedfx.pathwaystudio.com/app/search) to explore the molecular interactions and pathways concerning *PLA2R1* and its promoter methylation in breast cancer [26].

### 4.2. Patients and Tissue Samples

A total of 70 female patients with breast cancer (age range, 29–87; mean 57.2 ± 15.4 years) and 30 female patients with benign breast fibroadenomas (control) (age range, 25–63 years; mean 48.8 ± 11.6 years) were recruited from the Surgical Department of Kasr Alainy Teaching Hospital, Faculty of Medicine, Cairo University. The specimens were obtained for the period extending from March 2018 to September 2019 after obtaining approval of the Institutional Review Board (IRB # BC 2136, February 2018) in accordance with the Declaration of Helsinki. Written informed consent was obtained from all patients before they participated in this study. After surgical treatment, ranging from simple mastectomy to classical radical mastectomy, fresh sample cuts of the breast tumor lesions were excised and fixed with 10% neutral buffered formalin. Formalin-Fixed Paraffin-Embedded (FFPE) blocks were prepared for pathological evaluation and immunohistochemical detection in the pathology department of the Faculty of Medicine, Cairo University and stored at an appropriate temperature until use. All samples were confirmed as breast infiltrating ductal carcinomas and breast fibroadenomas using histopathological methods. None of the enrolled patients had received preoperative neo-adjuvant chemotherapy, immunotherapy or radiation therapy. We also excluded patients diagnosed with inflammatory breast cancer, with metastatic cancer or with a history of recurrent tumors.

### 4.3. Histological and Immunohistochemical Investigation

Histological grading, as well as the ER, PR, HER2 and Ki-67 status, were verified in the pathology department of the Faculty of Medicine, Cairo University. Tumor grading was performed according to the Modified Scarff–Bloom–Richardson–Elston–Ellis grading system (SBR-EE) [32]. All immunohistochemical analyses were conducted on routinely processed FFPE tissues. The immunostaining was performed using a BenchMark XT Ventana autostainer following the protocol instructions. The ER, PR and HER2 expressions were scored following the American Society of Clinical Oncology/ College of American Pathologists (ASCO/CAP) guidelines [33]. The expression of Ki-67 in breast tissue was studied by calculating the percentage of positively stained breast cancer cells [34]. For the molecular subtyping, immunohistochemical staining of ER, PR, HER2 and Ki-67 were used as surrogate markers to classify breast cancer tumors into luminal A, luminal B, HER2-positive and TNBC. The molecular subtypes were defined as follows—luminal A (ER+, PR+, HER2− and Ki-67 < 14%), luminal B (ER+, PR+ and HER2+) or (ER+, PR+, HER2- and Ki-67 ≥ 14%), HER2-positive (ER−, PR− and HER2+) and TNBC (ER−, PR− and HER2-) [16,17].

### 4.4. Molecular Biology Examinations

#### 4.4.1. RNA Extraction and Reverse Transcription

For the RNA extraction, a macro-dissection was performed on each sample and the total RNA was extracted from the FFPE breast tumor samples, after removing the paraffin with xylene using a RNeasy Fibrous Tissue kit (Qiagen, cat. No. 74704, Valencia, CA, USA) as described in the manufacturer’s protocol. We evaluated the purity and concentration of the RNA using a NanoDrop ND-100 Spectrophotometer (Thermo Scientific, Waltham, MA, USA).

Reverse transcription (RT) of the RNA was conducted using a miScript II RT kit (Qiagen, cat. No. 218161, Valencia, CA, USA) according to manufacturer’s instructions. The total reaction volume was 20 µL containing 100 ng of the total RNA that was reverse transcribed at 37 °C for 1 h, followed by inactivation of the reverse transcriptase at 95 °C for 5 min. A total of 30 ng of the newly synthesized complementary DNA (cDNA) served as a template for the quantification of PLA2R1 mRNA expression. The cDNA was diluted and stored at −80 °C until assayed.

#### 4.4.2. Quantitative Real-Time PCR (qRT-PCR)

The cDNA was amplified using quantitative real-time PCR. The amplification reactions were performed in 25 μL volumes containing 2.5 μL of diluted RT product, 2.5 μL from PLA2R1 mRNA Quanti Tect Primer Assay (Qiagen, cat. No. 249900, Valencia, CA, USA) for PLA2R1 mRNA assay, then nuclease-free water was added to reach the final volume. The following PCR cycling conditions were used 95 °C for 15 minutes, followed by 40 cycles at 94 °C for 15 s, 55 °C for 30 s and 70 °C for 30 s. The raw cycle threshold (Ct) values were collected using the (Rotor-Gene Q (Qiagen) Software 2.3.1.49). The mature PLA2R1 levels were normalized to the human Glyceraldehyde 3-phosphate dehydrogenase (GAPDH) levels and the experiment was performed in triplicate. By means of the ΔΔCt equation, the expression of PLA2R1 in malignant breast tissues was calculated in comparison with the fibroadenomas breast tissues using the endogenous control GAPDH.

#### 4.4.3. Genomic DNA Extraction and DNA Methylation

Genomic DNA was extracted from the FFPE breast tumor samples using a DNeasy tissue extraction kit (Qiagen, Cat. No. 69504, Valencia, CA, USA) according to the manufacturer’s instructions. The DNA purity and quantity were determined using a nanodrop (Thermo scientific, USA). After DNA extraction, a methylation assay was performed using EpiTect Methyl II PCR Assay (Qiagen, Valencia, CA, Cat. No. 335002) to assess the *PLA2R1* promoter average methylation level. Briefly, the genomic DNA was digested using the EpiTect Methyl II DNA Restriction Kit (Qiagen, Valencia, CA, Cat. No.335452) and real-time PCR was performed using RT SYBR Green ROX qPCR Mastermix (Qiagen, Valencia, CA, Cat. No 330520). All data were analyzed using the methylation assay software provided by Qiagen. The cycling PCR conditions involved 5 min at 94 °C, 40 cycles of 94 °C for 30 s, 72 °C for 60 s and 72 °C for 30 s.

### 4.5. Statistical Analysis

All statistical analyses were performed using the computer program Statistical Package for the Social Science (SPSS, Chicago, IL, USA) software version 15 for Microsoft Windows and GraphPad Prism 5.0 (GraphPad Software, CA, USA). The values were expressed as the mean ± standard deviation (SD) or median when appropriate. Levels of PLA2R1 mRNA expression were measured using RT-qPCR in the breast tissue specimens and normalized to GAPDH mRNA as reference gene. The fold change was calculated as ratio of PLA2R1 expression to the internal control. Of note, Log2 values were used to visualize the gene expression data for easier data interpretation. The Wilcoxon rank sum test was used to compare the malignant and benign breast tissues. The Kruskal–Wallis rank sum test and Dunn (1964) Kruskal–Wallis multiple comparison test were used to compare the levels of expression of PLA2R1 and its promoter methylation where they were depicted using boxplots. The correlations between the PLA2R1 expression and promoter methylation were evaluated using Spearman’s *r* correlation coefficient. ROC curve analysis was performed to assess the diagnostic and prognostic accuracy of PLA2R1 and *PLA2R1* promoter methylation and the AUC was also calculated [35]. Statistical significance was considered for *p* values lower than 0.05.

## Figures and Tables

**Table 1:** Clinico-pathological data of breast tissue samples included in the study.

Variable	*N* (%)
Benign fibroadenomas (control)	30 (30%)
Infiltrating ductal carcinomas	70 (70%)
**Tumor Grades**	
II	24 (34.2%)
III	46 (65.71%)
**ER Status**	
Negative	47 (67.14%)
Positive	23(32.85%)
**PR Status**	
Negative	47 (67.14%)
Positive	23 (32.85%)
**HER2 Expression**	
Negative	44 (62.8%)
Positive	26 (37.14)
**Molecular Subtypes**	
TNBC	25 (35.71%)
HER2-positive	22 (31.4%)
Luminal A	12 (17.14%)
Luminal B	11 (15.71%)

Abbreviations: ER: Estrogen receptor; PR: Progesterone receptor; HER2: Human epidermal growth factor receptor 2; TNBC: Triple negative breast cancer.

## Abbreviations

AUC	Area under the curve
ASCO/CAP	American Society of Clinical Oncology/ College of American Pathologists
CCLE	Cancer cell line encyclopedia
cDNA	Complementary DNA
c-MYC	Cellular myelocytomatosis
Ct	Cycle threshold
ER	Estrogen receptor
ESRRA	Estrogen-related receptor alpha1
FFPE	Formalin-fixed paraffin-embedded
GAPDH	Glyceraldehyde 3-phosphate dehydrogenase
HER2	Human epidermal growth factor receptor 2
JAK2	Janus kinase 2
PLA2R1	Phospholipase A2 receptor 1
PR	Progesterone receptor
qRT-PCR	Quantitative Real-time PCR
ROC	Receiver operating characteristic
RT	Reverse transcription
SBR-EE	Scarff-Bloom-Richardson-Elston-Ellis grading system
SD	Standard deviation
sPLA2	Secretory phospholipase A2
TNBC	Triple negative breast cancer
